# The Effect of Nutrition on Aging—A Systematic Review Focusing on Aging-Related Biomarkers

**DOI:** 10.3390/nu14030554

**Published:** 2022-01-27

**Authors:** Catarina Leitão, Anna Mignano, Marta Estrela, Margarida Fardilha, Adolfo Figueiras, Fátima Roque, Maria Teresa Herdeiro

**Affiliations:** 1 Institute of Biomedicine (iBiMED), Department of Medical Sciences, University of Aveiro, 3810-193 Aveiro, Portugal; anna.mig50@gmail.com (A.M.); martaestrela03@gmail.com (M.E.); mfardilha@ua.pt (M.F.); 2 Consortium for Biomedical Research in Epidemiology and Public Health (CIBERESP), 28001 Madrid, Spain; adolfo.figueiras@usc.es; 3 Health Research Institute of Santiago de Compostela (IDIS), 15706 Santiago de Compostela, Spain; 4 Research Unit for Inland Development, Polytechnic of Guarda (UDI-IPG), 6300-559 Guarda, Portugal; 5 Health Sciences Research Centre, University of Beira Interior (CICS-UBI), 6200-506 Covilhã, Portugal

**Keywords:** healthy aging, nutrition, cardiovascular disease risk, biomarkers

## Abstract

Despite the increasing life expectancy, an individual’s later years tends to be accompanied by a decrease in the quality of life. Though biological changes that occur through the natural process of aging cannot be controlled, the risk factors associated with lifestyle can. Thus, the main goal of this systematic review was to evaluate how nutrition can modulate aging. For this purpose, thirty-six studies were selected on (i) the efficiency of nutrition’s effect on aging, (ii) the evaluation of biomarkers that promote healthy aging, and (iii) how to increase longevity through nutrition, and their quality was assessed. The results showed that choosing low carbohydrate diets or diets rich in vegetables, fruits, nuts, cereals, fish, and unsaturated fats, containing antioxidants, potassium, and omega-3 decreased cardiovascular diseases and obesity risk, protected the brain from aging, reduced the risk of telomere shortening, and promoted an overall healthier life. With this study, the conclusion is that since the biological processes of aging cannot be controlled, changing one’s nutritional patterns is crucial to prevent the emergence and development of diseases, boost longevity, and, mostly, to enhance one’s quality of life and promote healthy aging.

## 1. Introduction

Aging is the gradual process of natural changes that occur throughout the human lifespan. This process begins in early adulthood; throughout the years, many mental and bodily functions begin to slowly decline, resulting in health issues, such as increased morbidity and decreased fertility [1,2,3]. Over the years, life expectancy has risen, with approximately 8% of the world population being over 65 years old, and in approximately 30 years, this number is expected to double [4]. However, this does not necessarily mean that people experience better health in their later years when compared with other generations [5,6].

In fact, despite the life expectancy increase, aging is a mechanism that has multifaceted features that are linked: molecular, cellular, physiological, and functional levels that ultimately lead to chronic diseases [7]. At the molecular level, aging is represented by genomic instability, telomere attrition (as they tend to shorten), epigenetic alterations, loss of proteostasis, deregulated nutrient sensing, stem cell exhaustion, and altered intercellular communication, which are believed to be decisive to determine one’s lifespan [8]. These risk factors might impact the cellular level by deregulating signaling; thus, causing cellular senescence and mitochondrial dysfunction [9]. Consequently, the physiological and functional levels weaken, triggering the development of chronic inflammation, alterations in energy metabolism, such as variations in insulin sensitivity, undermining neuronal [10], and sensory functions, mainly visual, auditory, and touching (or movement) [11,12]. On a physical level, hair normally turns white, thinner, and takes longer to grow, and the skin becomes less elastic and more wrinkled, as a result of less efficient vitamin D synthesis [13]. Moreover, there are some factors that are usually associated with specific disorders that might appear: hyperglycemia, hypercholesterolemia, and hypertension [14]. Altogether, these effects give rise to numerous age-associated disorders that result from these alterations, such as sarcopenia, represented by the decline in skeletal muscle mass, and, therefore, weakening physical functioning, which is derived from chronic inflammation, hormonal changes, cell dysfunction, an unhealthy diet, lack of physical activity [15], neurodegenerative diseases, heart diseases, diabetes, and cancer [16,17].

Despite the biological changes associated with aging, there are risk factors, such as lifestyle and dietary patterns [18,19], which, if altered, can promote healthy aging, and are characterized by the development and maintenance of the functional abilities that enable one’s wellbeing [20]. Healthy lifestyles promote the maintenance of cognitive abilities and boost the immune system [18,19,21,22]. Studies show that adoption of a Mediterranean diet, characterized by a decreased consumption of saturated animal fat and red meat and a higher intake of fruits and vegetables, along with maintaining a healthy weight and reducing salt intake [23,24,25,26], and lifestyle changes, such as engaging in physical and social activities and reducing smoking and alcohol consumption, can significantly modulate the inflammatory state of the body. Consequently, general health improves throughout the diverse stages of life [23,27,28]. It is also believed that these key factors may be viewed from a life-course perspective since a change of these routines can shape the rate at which human cells or organs enter in senescence, delay the onset of chronic diseases, and promote mobility, mental function, and wellbeing [23,29]. However, the main “gaps” remain as to what types of diets or what food components could actually improve healthy aging, and what biomarkers are modulated by the diet and associated with chronic diseases, in order for them to be considered more when evaluating or preventing the incidence of these diseases.

To address these gaps, the main goal of this systematic review was to perceive the quantity and quality of different diets or aspects in nutrition, how they could modulate biomarkers and prevent aging-related diseases, in order to enlighten new intervention strategies. Biomarkers that are linked to aging-associated metabolism, inflammation processes, cognitive decline, and telomere attrition were scrutinized in order to understand how these mechanisms could actually influence healthy aging. Moreover, it could provide information to future health professionals.

## 2. Materials and Methods

### 2.1. Protocol and Registration

This systematic review followed the PRISMA statement guidelines, and it was recorded in the international database of prospectively registered systematic reviews (PROSPERO)—registration number CRD42021244473 [30].

### 2.2. Search Strategy and Inclusion Criteria

For this systematic review, searches were conducted in PubMed scientific databases on 28 February 2021.

The search was conducted by two independent researchers, and it was primarily designed to spot relevant studies on the efficacy of nutrition’s effect on aging, on the evaluation of molecules that promote healthy aging, and on how to increase longevity through nutrition. The following keywords and their equivalents were used in PubMed:

“(diet* OR nutri*) AND (age OR ageing OR aging OR old OR older OR elder*) AND (marker OR biomarker)”

The selection criteria applied in this review were the following: (1) language: documents had to be published in English; (2) published studies from 1 January 2010 to 28 February 2021; (3) participants/population: adults/humans; (4) types of studies to include: randomized and non-randomized trials (including clusters—randomized and clusters—non-randomized), and observational studies (including case control, cross sectional, cohort, before and after, and interrupted time series). Study protocols, reviews, systematic reviews, and meta-analysis were excluded. The included studies assessed the importance of a healthy diet in order to ensure healthy aging through the study of the molecules involved. All studies that mentioned this impact were considered.

All titles retrieved from database were reviewed independently; the inclusion and exclusion criteria were applied by two independent researchers (A.M. and C.L.) and validated by a third researcher (M.E.) in cases where there was no consensus.

### 2.3. Quality Assessment of the Included Studies

The quality assessment of the included studies involves using five criteria [31]: (1) study group allocation: random: 2; quasi-random: 1; selected controls: 0; (2) allocation unit: cluster (for example, practice): 2; physicians/physiotherapists/researchers: 1; patients: 0; (3) baseline differences: presence of baseline differences with statistical adjustments: 2; baseline differences with no statistical adjustments: 1; absence baseline differences: 0; (4) objectivity of the result: blinded assessment: 2; no blinding but defined assessment criteria: 1; no blinding and poorly defined: 0; (5) completeness of follow-up: ≥90%: 2; 80–90%: 1; <80% or not described: 0.

All studies were scored from 0 to 10, based on the sum of the scores for each parameter and it was evaluated by two independent researchers (A. M. and C. L.). Higher scores indicate higher quality research.

### 2.4. Data Extraction and Analysis

The articles that met the inclusion criteria were summarized in a table for: author and publication year; study design, country, setting, study population, sample size, participants’ characteristics, and the main results and involved biomarkers. Two independent researchers (A.M. and C.L.) extracted data and compared their findings. In cases of disagreement, a third reviewer (M.E.) served as a referee to help reach an agreement. 

## 3. Results

### 3.1. Study Selection

**Figure 1:**
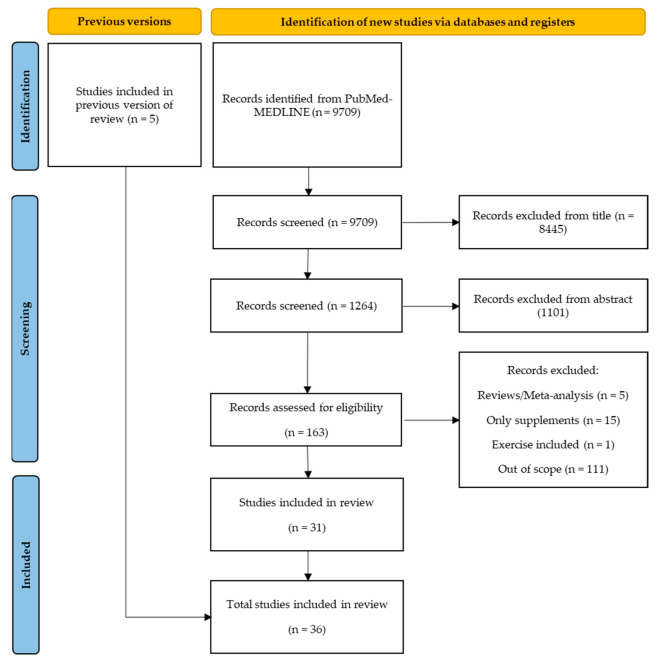
Application of search strategies to retrieve the total number of studies for analysis.

The search strategy involved retrieving 9709 citations from PubMed–MEDLINE database, the eligible articles were carefully chosen based on the title and abstract. In the first version of this systematic review, five studies were selected. Despite these studies not appearing in the chosen string of keywords, they were present in the bibliography of the studies identified from the database. Those studies were first selected on the title, resulting in 1264 studies, and after selection based on the abstract, 163 studies were analyzed. These 163 studies were evaluated based on their full-texts, in which 31 were deemed suitable for inclusion in the current review [32,33,34,35,36,37,38,39,40,41,42,43,44,45,46,47,48,49,50,51,52,53,54,55,56,57,58,59,60,61,62,63,64,65,66,67] (Figure 1). 

### 3.2. Quality Assessment

The quality of all of the included studies was assessed [31]. The median score for all of the studies was 6/10. Seven studies received a score of 4 or less out of 10 [38,48,52,55,58,64,66]. Fifty-eight percent of the studies had a total score of 5–7 out of 10 [32,33,35,36,39,40,41,42,44,46,47,49,51,53,54,56,57,61,62,63,65]. Eight studies received a score of 8 or higher out of 10 [34,37,43,45,50,59,60,67]. The results are presented in Supplementary Material (Table S1).

### 3.3. Study Characteristics

Table 1 summarizes the study characteristics of the included papers. This table presents information from the included articles on the study design, country, setting, study population, sample size, participants’ characteristics (age, gender, body mass index (BMI), and if they had any disease), and data collection procedures. 

#### 3.3.1. Study Design

A total of 12 of the 36 included studies were randomized controlled trials [33,34,37,41,43,50,56,57,59,60,61,67], 8 were cross-sectional studies [36,39,40,44,45,49,51,58], 6 were prospective cohort studies [32,47,55,63,64,65], 4 were cohort studies [46,52,62,66], 2 were crossover studies [38,53], 2 were randomized non-controlled trials [35,42], and 2 were prospective non-randomized: pre/post studies [48,54].

#### 3.3.2. Location

Most studies (55.6%) were conducted in Europe [32,34,37,38,40,41,42,43,44,46,47,49,50,54,56,58,59,61,62,63]. Nine studies (25%) took place in North America [35,36,39,52,53,55,57,64,66], six were undertaken in Asia [33,45,48,51,60,65], and one study was carried out in Australia [67].

#### 3.3.3. Setting

The majority of studies (61.1%) were conducted in academic medical centers [32,36,37,39,40,44,46,47,48,49,51,52,53,54,55,57,59,60,62,63,66,67]. Fourteen of the thirty-six interventions (38.9%) occurred in the context of hospital care [33,34,35,38,41,42,43,45,50,56,58,61,64,65], with nine of them in university hospitals [35,38,41,42,56,58,61,64,65].

#### 3.3.4. Sample Size and Study Population

Twelve of the thirty-six (33.3%) included studies presented a sample size under 100 participants [33,35,38,41,42,48,52,53,56,60,61,67], thirteen studies (36.1%) had a sample size between 100 and 1000 participants [32,34,37,43,45,50,51,54,55,57,59,62,65], nine (25%) between 1000 and 10,000 [36,39,44,46,47,49,58,64,66], and two had more than 10,000 participants [40,63].

Twenty two of the thirty-six included studies examined the results based on the adult population [32,33,35,36,37,40,46,47,48,49,52,53,54,55,57,58,59,60,63,64,66,67] and fourteen only recruited older adults [34,38,39,41,42,43,44,45,50,51,56,61,62,65].

All of the studies were based on age and gender (both genders, only men or only women) to select the participants. Six of the thirty-six studies (19.4%) included participants with type-1 diabetes (T1D), type-2 diabetes (T2D), mild cognitive impairment (MCI), and cardiovascular disease (CVD), or who were at risk of suffering from these diseases [34,35,37,43,50,53,55], and half of the studies had specific BMI [35,36,37,41,42,44,48,52,54,55,56,57,59,60,61,62,65,67] as inclusion criteria. 

#### 3.3.5. Influence of Different Types of Diet on Biomarkers

All of the studies aimed to evaluate the nutritional state, as described in our main goal. Besides the nutritional state, eight of the studies also considered telomere length [32,36,37,39,58,62,64,66] and five of the studies evaluated nutritional state effects in cognitive decline [33,50,52,53,65]. The main molecular outcomes and the tested biomarkers are described in Table 2.

**Figure 2:**
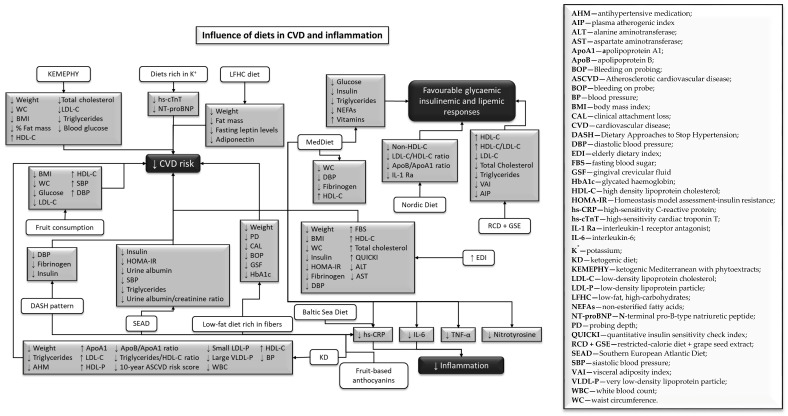
Influence of diets in cardiovascular diseases hallmarks and inflammation.

**Figure 3:**
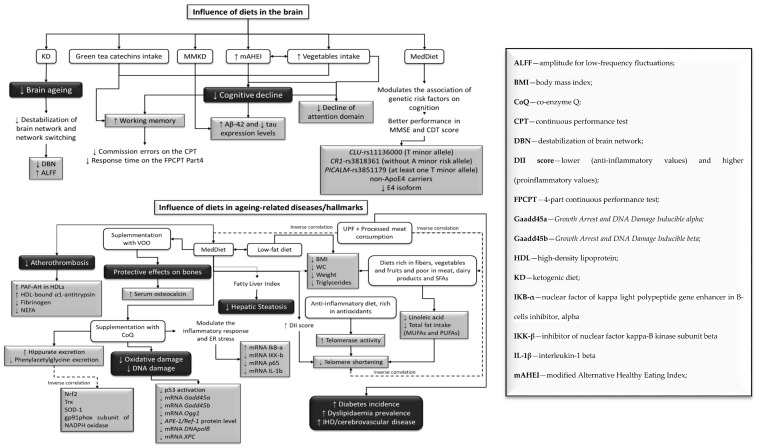
Influence of different diets in brain aging (up) and in aging-related disease/hallmarks (down).

The ketogenic diet (KD) reduced CVD risk by improving CVD biomarkers (Figure 2) in T2D patients and decreasing inflammation [55]. It also prevented brain aging (Figure 3) by avoiding the destabilization of brain network and network switching [52]. Network stability is represented by the ability of the brain to maintain functional communication between its regions, and when this process is affected, network switching occurs from the largescale reorganization of the network modules. Moreover, the Mediterranean diet (MedDiet) regulated the relationship between genetic risk factors and cognition [50], protecting participants with specific variants of the *CR1* gene (without A minor allele of the rs3818361), the *CLU* gene (with T minor allele of the rs11136000), the *PICALM* gene (with T minor allele of the rs3851179), and both non-ApoE4 and ApoE4 carriers. Variations in this diet, such as the modified Mediterranean–ketogenic diet (MMKD), prevented cognitive decline (Figure 3) in adults with Alzheimer’s disease (AD) risk, by increasing amyloid-beta 42 (Aβ42) and decreasing tau expression [53]. 

The modified Alternative Healthy Eating Index (mAHEI) was used to assess diet quality in an older Chinese population, considering consumption of fruits, vegetables, soy protein, fish/meat/eggs, whole grain, fried foods, and alcohol [65]. In this study, a high diet quality combined with a varied vegetable intake was linked to a lower risk of cognitive decline (Figure 3). Daily intake of green tea catechins demonstrated beneficial effects on working memory in adults, by decreasing the commission errors in the continuous performance test (CPT), and the time response in the 4-part CPT (FPCPT) [33]. 

The MedDiet demonstrated beneficial effects in several key points. The role of the dietary inflammatory index (DII) used this diet in telomere shortening (Figure 3) [37]. An anti-inflammatory diet, rich in antioxidants, could reduce the rate of telomere shortening, by improving telomerase activity [62] in individuals at high risk of CVD [37,39,62,66]. In addition, diets rich in fibers, vegetables, and fruits, and poor in meat, dairy products, and PUFAs, as well as waist circumference (WC) reduction, are beneficial in improving leukocyte telomere length (Figure 3) [39,58,64]. In contrast, consumption of ultra-processed foods (UPFs) and processed meat was negatively associated with MedDiet adherence, and it was positively related with a higher risk of having shorter telomeres (Figure 3) [32,36,63]. UPF was also linked to an increased risk of CVD, ischemic heart disease (IHD)/cerebrovascular disease, and all-cause mortality (Figure 3) [63]. 

Cardiovascular risk factors (Figure 2) were also attenuated in the presence of a dietary approach to stop hypertension (DASH) pattern, decreasing the levels of fibrinogen, insulin, and diastolic blood pressure (DBP) [45]. In contrast to a Western diet-like high-fat (WDHF) and a Western diet-like high-carbohydrate (WDHC), MedDiet meals promote positive effects on glycemic insulinemic, and lipemic responses (Figure 2), due to lower levels of glucose, insulin, triglycerides, and non-esterified fatty acids (NEFAs) [56]. The ketogenic–Mediterranean diet, supplemented with phytoextracts (KEMEPHY), also led to reductions in weight, BMI, WC, % fat mass, total cholesterol, LDL-C, triglycerides, and blood glucose, and increases in HDL-C [54].

A healthy Nordic diet reduced non-high-density lipoprotein cholesterol (non-HDL-C), low-density lipoprotein cholesterol (LDL-C)/HDL-C ratio, apolipoprotein B (ApoB)/apolipoprotein A1 (ApoA1) ratio, and interleukin-1 receptor antagonist (IL-1 Ra), improving lipid profile (Figure 2), and having a beneficial effect on low-grade inflammation [59]. The Southern European Atlantic diet (SEAD), a common diet in the Iberian Peninsula, enabled reduced concentrations of C-reactive protein (CRP), triglycerides, and insulin, which could help prevent myocardial infarction [40]. Diets rich in potassium intake attenuated CVD risk (Figure 2) since they prevented cardiomyocyte injury, through high-sensitivity cardiac troponin T (hs-cTnT) and cardiac dysfunction, through N-terminal pro-B-type natriuretic peptide (NT-proBNP) [49]. A restricted-calorie diet (RCD), allied to daily consumption of grape seed extraction (GSE), improved blood lipid profile and helped ameliorate some CVD risk factors (Figure 2), by reducing LDL-C, total cholesterol, the triglycerides visceral adiposity index, and the plasma atherogenic index. Moreover, it promoted higher levels of HDL-C and HDL-C/LDL-C ratio in obese or overweight adults [60]. A low-fat high-carbohydrate (LFHC) diet reduced weight, fat mass, fasting leptin, and adiponectin levels, when compared to a moderate-fat diet (18% protein, 36% fat, 46% carbohydrate) [57]. 

In older adults, a low-fat diet rich in fibers, improved periodontal disease markers, such as clinical attachment loss (CAL), bleeding on probe (BOP), probing depth (PD), and gingival crevicular fluid (GSF), as well as on the metabolic profile (body weight, hs-CRP and glycated hemoglobin) [48].

A modified version of MedDiet score, Elderly Dietary Index (EDI), which evaluates elderly adherence to dietary recommendations, was associated with reduced weight, BMI, WC, insulin, fibrinogen, diastolic blood pressure, homeostasis model assessment-insulin resistance (HOMA-IR), alanine aminotransferase (ALT), aspartate aminotransferase, and higher HDL-C concentrations, fasting blood sugar, total cholesterol, and quantitative insulin sensitivity check index (QUICKI) [51]. 

Fruit consumption, both in fruit or in juice, reduced several cardiometabolic risk factors (Figure 2), such as BMI, WC, glucose levels, and LDL-C in elders with metabolic syndrome [44]. Fruit-based anthocyanins also mitigated the negative effects of a high fat high energy (HFHE) diet, by decreasing inflammatory biomarkers, such as CRP and interleukin-6 (IL-6) [67].

MedDiet also decreased hepatic steatosis risk, based on the fatty liver index [47]. A low-fat diet and MedDiet decreased weight, BMI, WC, and triglyceride levels, in adults with T1D and metabolic syndrome (MetS) [35]. MedDiet also decreased atherothrombosis biomarkers (Figure 3), such as platelet activating factor acetylhydrolase (PAF-AH), HDL-bound α1-antitrypsin, fibrinogen and NEFAs in older adults with high cardiovascular risk [43]. In older adults, MedDiet supplemented with coenzyme Q10 (MedDiet + CoQ) promoted higher excretion of urinary metabolites, such as hippurate (Figure 3) related to oxidative stress, in comparison with a saturated fat (SFA) diet [38]. The MedDiet + CoQ also positively modulated the inflammatory response and ER stress [61], by decreasing p65 (protein involved in NF-κB heterodimer formation, nuclear translocation and activation), inhibitor of nuclear factor kappa-B kinase (IKK-b) and interleukin-1b (IL-1b) mRNA levels. Moreover, it protected DNA from oxidative damage, by reducing the activation of p53, and, consequently, downregulated the expression of genes *Gadd45a*, *Gadd45b*, *OGG1*, *APE1/Ref-1*, *DNA polβ*, and *XPC*, all linked to p53-dependent DNA repair [41,42].

A Nordic alternative to the MedDiet, the Baltic Sea Diet, was evaluated regarding inflammation markers (Figure 3), and a reduction of high-sensitivity C-reactive protein (hs-CRP) was noted [46]. Consumption of a MedDiet supplemented with virgin olive oil (MedDiet + VOO) demonstrated protective effects on the bones (Figure 3), by increasing serum osteocalcin [34].

## 4. Discussion

This systematic review was accomplished to, first, demonstrate how different diets or different aspects in nutrition impact aging-related diseases, and second, to pinpoint putative molecular markers that change with diet and nutrition along aging. The results of the studies analyzed in this systematic review show that avoiding unhealthy habits, mainly concerning diets, may improve life quality and promote healthy aging (Figure 2 and Figure 3).

The diets studied in this systematic review presented diverse similarities. KD is a low-carbohydrate diet, whereas MedDiet is based on low consumption of saturated animal fat and red meat, and a high intake of fruits, vegetables, fish, olive oil, nuts, and vitamins [68]. The DASH diet pattern is very similar to MedDiet, since it is rich in the same products and poor in SFAs, added sugars, sodium, and refined carbohydrates [69]. The KEMEPHY diet maintains the use of vegetables and olive oil just like the MedDiet [70], but presents elevated ketone bodies in the blood or urine [71]. The SEAD is represented by high intakes of fresh fish, red meat, and pork products, legumes and vegetables, vegetable soup, potatoes, dairy products, whole-grain bread, and wine [40]. The Baltic Sea diet is described as a variant of the MedDiet with healthy Nordic foods [72]. The healthy Nordic diet is based on the consumption of whole-grain products, abundant intake of berries, fruits, and vegetables, rapeseed oil, three fish meals per week, low-fat dairy products, and a reduction of sugar-sweetened products [59]. The MMKD is a very low-carbohydrate diet that is modeled on a MedDiet, emphasizing protein sources low in saturated fat (fish, lean meats), healthy fats, fruit and vegetable consumption, whole grains, and a glass of wine per day [53].

The adoption of a KD reduced CVD risk, by improving CVD biomarkers (Figure 3) in T2D patients, and decreased inflammation [55,73,74,75]. Since inflammation has a role in every facet of CVD pathogenesis [76] and hs-CRP and WBC count are well-established markers of inflammation and risk factors for CVD [77,78], the decrease of these markers in the CCI indicated that inflammation was diminished after 1 year of intervention. KD also protected brain aging (Figure 2) by avoiding the destabilization of the brain network, which is what happens when glucose is used as fuel [52]. These stabilizing effects of nutritional ketosis were replicated when exogenous ketones were administrated, suggesting that they were specifically due to glucose metabolism rather than ketone body metabolism [52]. Furthermore, it supports the hypothesis that there are some beneficial neural effects associated with the hypocaloric state; intermittent fasting and severe caloric restriction may result from the use of ketone bodies by the brain [79].

The MedDiet modulated the impact of genetic risk factors on cognition (Figure 3) [50]. The variants studied have been associated as risk factors for AD [80,81,82] and cognitive decline, mainly with poor memory performance in population-based studies [83,84]. The MedDiet has already been revealed as protective in cognitive functioning [85], cognitive decline [86], and the development of mild cognitive impairment and dementia [87,88]. Daily intake of green tea catechins also demonstrated beneficial effects on the working memories in adults [33,89,90]. The MMKD prevented cognitive decline (Figure 3) in adults with Alzheimer’s disease (AD) risk, by increasing Aβ42 and decreasing tau expression [53]. Moreover, a high diet quality that prioritizes foods that are the base of the KD and MedDiet, allied to a diverse vegetable intake, was linked to a decrease of cognitive decline risk [65]. This was supported by previous studies that demonstrated that dietary interventions had a huge impact on cognitive deterioration [91]. Additionally, this protective effect may be due to individual characteristics of foods. Antioxidants and phenolic compounds can minimize oxidative damage and inflammation, and boost neuronal antioxidant defenses [92,93].

The MedDiet was used to assess the role of DII in telomere shortening, proving that an anti-inflammatory diet or one rich in antioxidants could reduce the rate of telomere shortening in individuals at a high risk of CVD [37]. Studies that evaluated adherence to the MedDiet achieved the same conclusions [39,62,66,94,95,96,97]. 

Higher consumptions of vegetables [58] and diets rich in fibers [39,64] were also positively associated with leukocyte telomere length, as well as lower WC, whereas SFAs were negatively linked [58]. Thus, an anti-inflammatory diet can avoid telomere shortening and, consequently, reduce the impact of aging and cancer development [98]. In contrast, consumption of UPF and processed meat was linked to an increased risk of having shorter telomeres [32,36]. UPFs are industrial formulations of food-derived substances (oils, fats, sugars, starch, protein isolates) that include flavorings, colorings, emulsifiers, and other cosmetic additives [32]. UPFs have been linked with several diseases, such as hypertension [99], obesity [100], MetS [100], T2D [101], and cancer [101]. Higher total intakes of salt, saturated fat, and sugar, as well as inadequate intakes of fiber and minerals, could explain the association between UPF consumption and the risk of developing short telomeres. [102].

The MedDiet [35,47,56], or its components [44,67], RCD, allied to daily consumption of GSE [60], DASH patterns [45], LFHC diet [57], the Baltic Sea diet [46], the Nordic diet [59], SEAD [40], the KEMEPHY diet [54], or the use of EDI scores to assess MedDiet adherence [51], promoted decreased inflammatory markers and improved cardiometabolic risk factors (Figure 2), including components that belong to MetS. MetS is one of the most significant risk factors associated with CVD [103,104] and it is defined as having at least three of the following characteristics: (i) central obesity, (ii) increased serum triglyceride levels, (iii) low HDL levels in the blood, (iv) cholesterol levels, (v) hypertension, and (vi) higher fasting blood glucose levels [104]. Since these diets are very similar, this might explain the similar results. In several studies, researchers note that the MedDiet is able to prevent CVD [24,26,68,105]; this effect is due to the combination of different foods that have anti-inflammatory properties. The DASH diet pattern was already established as a potential treatment for hypertension [106], to reduce both fasting and postprandial insulin concentrations [107], and CRP levels [108,109]. The KEMEPHY diet, by inducing a physiological ketosis through elevated ketone bodies present in blood or urine [71], stimulates positive changes in cardiovascular risk factors and body composition [110].

SEAD, just like the MedDiet, is rich in folate and vitamin C from vegetables, as well as omega-3 fatty acids from fish; these compounds have already been connected with reduced insulin [111,112] and low hs-CRP [113]. This inflammatory marker was also reduced in this diet, suggesting that it may reduce CVD risk (Figure 2). The Baltic Sea diet also promotes the reduction of CRP concentrations (Figure 2), due to the presence of Nordic fruits and berries and cereals [46]. They contain antioxidant components, such as polyphenols, minerals, vitamins, and dietary fibers, which diminish these levels and the release of E-selectin [114,115]. This molecule is produced and released into circulation during endothelial injury, and its reduction improves endothelial function [115]. The healthy Nordic diet decreases IL-1 Ra [59], which is one of the most sensitive inflammatory markers, in obesity and MetS. It has been associated with high intakes of SFAs and lower consumption of berries, fruits, and whole-grain products [116,117,118]. Moreover, higher levels of IL-1 Ra have been revealed to predict T2D and the progression of MetS to T2D [116]. In addition, this healthy Nordic diet promotes the reduction of non-HDL-C and LDL-C, which are designated to have an important impact on CVD morbidity and mortality [119,120]. CVD risk is also attenuated by high intakes of potassium (Figure 2) due to its nonlinear association with CVD biomarkers (Figure 2) [49,121,122], which is supported by previous studies [123,124,125], thus endorsing the World Health Organization (WHO) recommendation that adults increase their potassium intake [126]. In contrast, CVD, IHD/cerebrovascular disease, and all-cause mortality was linked to UPF consumption [63,127,128,129].

The MedDiet also decreases risk factors associated with other comorbidities (Figure 3), such as hepatic steatosis [47], atherothrombosis [43], oxidative stress, by promoting the excretion of urinary metabolites [38], and, allied to virgin olive oil, demonstrates protective effects on bone tissue, by increasing serum osteocalcin [34]. 

The MedDiet, allied to a supplementation of CoQ, was shown to protect DNA from oxidative damage (Figure 3) by reducing the activation of p53 [41] and, consequently, the expression of genes involved in p53-dependent DNA repair were downregulated [42]. Previous studies demonstrated that DNA damage plays an important role in the pathogenesis of atherosclerosis (buildup of fats, cholesterol, and other substances in and on the artery walls), as well as other aging-related diseases [130]. As for diets rich in PUFAs—CoQ supplements have been shown to mitigate oxidative and lipid peroxidation chain reaction damage [131,132], regenerate other antioxidants, such as α-tocopherol and ascorbate [133,134,135], and reduce CVD risk and inflammation [131]. The fatty acid content of cellular membranes and fluids is linked to oxidative stress, so it was expected that MedDiet + CoQ helped prevent this event [136,137,138]. Furthermore, recent research has shown that changes in the expression of p53 and the p53-regulated DNA damage response genes, such as *gadd45a* and *mdm2*, can be used as genotoxic and carcinogenic stress markers [139,140]. This diet also has an additional influence on the inflammatory response and ER stress [61], since it reduces the p65, IKK-b, and IL-1b gene expression.

A low-fat diet rich in fibers has demonstrated protective effects on periodontal disease (Figure 3), by reducing its biomarkers in older adults [48]. This disease has already been associated with obesity [109,141]. Other studies reported on how weight reduction promotes a decrease in system inflammation, as well as a loss of adipose tissue [142], and since hs-CRP was decreased in this study, obesity and periodontal disease may be connected by inflammation [142].

In contrast, previous studies [143,144,145,146,147,148,149] have demonstrated that unhealthy habits, such as westernized diets (high intakes of red meat, processed foods, ‘‘fast-foods’’, high-fat dairy products, snacks, and sugary soft drinks, and low intakes of fruits, vegetables, vitamins, and minerals), lack of physical activity, alcohol consumption, or smoking can potentiate the risk of development of diabetes, obesity, cardiovascular diseases, and cancer. Thus, even in the absence of traditional CVD risk factors (smoking, T2D, high BP, and elevated levels of cholesterol), weight-loss strategies should always be recommended in order to keep a healthy body weight to decrease CVD and other comorbidities risk.

Despite the insights provided in this systematic review, there is still much information that needs further research. Designing proper diets and addressing new strategies to combine them with different types of physical activity should be further explored. Moreover, extending this type of research to the cancer field may help in the discovery of new biomarkers that can promote an early diagnosis and treatment or novel approaches to prevention

This systematic review has several strengths, specifically, the extensive and systematic research of articles on the stated theme and information concerning the different effects of multiple diets. This study sought to compact different diets and food components that help decrease the prevalence and severity of several diseases, such as cardiovascular diseases, obesity risk, brain diseases, and even, delay the progression of cancer, through the reduction of telomere shortening. These results are of major importance to the development of new interventions in nutrition targeting aging-related biomarkers. However, this systematic review also presents some limitations. One of the limitations is the use of only one database (PubMed), which may have resulted in a failure to consider other potentially relevant articles on other databases. Another limitation was the difficulty to organize and structure the types of diets and biomarkers, since they present different effects. Finally, some of the biomarkers analyzed in this systematic review are not always valid for certain health outcomes, since they can be considered subrogated variables.

## 5. Conclusions

In conclusion, this systematic review demonstrated the necessity for individuals to improve their diets, to reduce the emergence and development of several comorbidities and promote healthy aging. Diets rich in vegetables, fruits, nuts, cereals, fibers, fish, unsaturated fats, containing antioxidants, vitamins, potassium, omega-3—and reducing red meat and UPF intake—could prevent obesity, CVD, and inflammation, and promote favorable glycemic, insulinemic, and lipidemic responses. Moreover, the MedDiet and KD, or a combination of these diets (MMKD), and increasing consumption of vegetables and green tea catechins, could improve one‘s working memory and decrease destabilization of the brain network and the attention domain, preventing cognitive decline. Finally, the MedDiet, supplemented with CoQ or VOO, or a low-fat diet, also rich in antioxidants, could help to decrease the prevalence of atherothrombosis, hepatic steatosis, diabetes, and telomere attrition, as well as prevent oxidative and DNA damage. These diets can enhance one‘s quality of life and increase life expectancy. Moreover, a putative panel of molecular markers (see Figure 2 and Figure 3) would follow the impact of diet/nutrition alterations during aging.

## Figures and Tables

**Table 1:** Synthesis of the studies’ characteristics.

Authors (Year)	Study Design	Country	Setting	StudyPopulation	Sample Size (N)	Participants Characteristics	Data Collection Procedure
Alonso-Pedrero, Lucia et al., 2020 [32]	Prospective Cohort Study (PCS)	Spain	Academical Medical Center (AMC)	Adults	886	Age (A)—≈67.7 years Gender (G)—72.8% men	Telomere length (TL) was measured using saliva samples and ultra-processed food (UPF) consumption was collected using a validated 136-item food frequency questionnaire (FFQ); the association between consumption of energy-adjusted UPF and the risk of having short telomeres was evaluated using logistic regression models.
Baba, Yoshitake et al., 2020 [33]	Randomized Controlled Trial (RCT)	Japan	Hospital Care (HC)	Adults	52	A—50–69 years G—50% men	For 12 weeks, participants took either (1) three placebo capsules or (2) three catechin capsules per day. At baseline and at 12 weeks after ingestion, blood biomarkers, the Mini-Mental State Examination Japanese version (MMSE-J), and hematologic tests were measured. Body weight (BW), hazard ratios (HR), systolic blood pressure (SBP), and diastolic blood pressure (DBP), as well as the Cognitrax test battery were measured at baseline, after a single dose, and after 12 w of daily ingestion.
Fernández-Real, José Manuel et al., 2012 [34]	RCT	Spain	HC	Elders	127	A—55–80 years G—men Disease (D)—Type 2 Diabetes (T2D) or cardiovascular disease (CVD) risk	Participants were randomized to three intervention groups: (1) Mediterranean Diet (MedDiet) + virgin olive oil (VOO); (2) MedDiet + nuts; and (3) low-fat diet (control). Dietary intakes were accessed by a 137-item FFQ. Glucose, total cholesterol (TC), high-density lipoprotein cholesterol (HDL-C), triglycerides, fasting insulin, total osteocalcin (TOC), undercarboxylated osteocalcin (UOC), and C-telopeptide of type I collagen (CTX) and procollagen I N-terminal propeptide (P1NP) levels were measured.
Fortin, A. et al., 2018 [35]	Randomized Trial (RT)	Canada	University Hospital (UH)	Adults	28	A—18–65 years G—57% men Body mass index (BMI) ≥ 25 kg/m^2^ D—Type 1 Diabetes (T1D) ≥ 12-month	For 6 months, participants were randomly assigned, randomized into two intervention groups: (1) MedDiet or (2) low-fat diet. Anthropometric (waist circumference WC), metabolic, and nutritional analyses were performed at inclusion, 3 months, and 6 months.
Fretts, Amanda M. et al., 2016 [36]	Cross-sectional study (CSS)	USA	AMC	Adults	2846	A—39.6 ± 16.4 years G—60.2% women BMI—32 ± 8 kg/m^2^	A 119-item FFQ was used to assess dietary factors, such as past-year consumption of processed meat and unprocessed red meat. Leukocyte telomere length (LTL) was determined using quantitative polymerase chain reaction (qPCR). Associations of intake of processed meat and unprocessed red meat with LTL were estimated by generalized equations.
García-Calzón, Sonia at al., 2015 [37]	RCT	Spain	AMC	Adults	520	A—67.0 ± 6.0 years G—55% women BMI > 25 kg/m^2^ D –T2D or high CVD risk	LTL was measured by quantitative real-time (qRT)-PCR and dietary inflammatory index (DII) was calculated using self-reported data collected via the questionnaire.
González-Guardia, Lorena et al., 2015 [38]	Cross-over study (COS)	Spain	UH	Elders	10	A ≥ 65 years G—50% men	For 4 weeks, participants followed four different isocaloric diets: (1) MedDiet supplemented with coenzyme Q10 (Med + CoQ) diet; (2) MedDiet; (3) Western diet rich in saturated fatty acids (SFAs) diet; (4) Low-fat high-carbohydrate (LFHC) diet enriched in n − 3 polysaturated fatty acids (PUFAs). Urine samples were collected for nuclear magnetic resonance (NMR) spectroscopy at baseline and after a 12-h fast (postintervention).
Gu, Yian et al., 2015 [39]	CSS	USA	AMC	Elders	1743	A— ≥ 65 years G—68.3% women	The MedDiet was calculated from collected data from FFQ. LTL was retrieved from leukocyte DNA using a RT-PCR to calculate ratio of telomere to single-copy gene sequence (T/S ratio).
Guallar-Castillón, Pilar et al., 2012 [40]	CSS	Spain	AMC	Adults	10,231	A ≥ 18 years G—51.6% women	A validated computerized diet history was used to assess the diet. Southern European Atlantic Diet (SEAD) adherence was assessed using a nine-food component index. C-reactive protein (CRP), uric acid, TC, low-density lipoprotein cholesterol (LDL-C), HDL-C, triglycerides, glucose, glycated hemoglobin, insulin, leptin, and fibrinogen levels were measured in 12 h fasting blood samples, while creatinine and albumin were measured in urine.
Gutierrez-Mariscal, Francisco M. et al., 2012 [41]	RCT	Spain	UH	Elders	20	A— ≥ 65 years G—50% men BMI—20–40 kg/m^2^	Three isocaloric diets were followed for a 4-week each: (1) MedDiet, (2) Med + CoQ diet, and (3) SFA diet. mRNAs levels for p53, p21, p53R2, and mdm2 were determined.
Gutierrez-Mariscal, Francisco M. et al., 2014 [42]	RT	Spain	UH	Elders	20	A ≥ 65 years G—50% men BMI—20–40 kg/m^2^	Three different diets for 4 weeks: (1) Med + CoQ diet, (2) MedDiet, and (3) SFA diet. Metabolic levels, food intake, growth arrest and DNA damage inducible alpha (*Gadd45a)* and beta *(Gadd45b)* gene expression and protein levels, and p53 inducible targets for DNA repair were measured.
Hernáez, Álvaro et al., 2020 [43]	RCT	Spain	HC	Elders	358	A—55–80 years G—63% women D—T2D or CVD risk	Three interventions: (1) MedDiet-VOO (received 1 L/w of virgin olive oil); (2) MedDiet-Nuts (210 g/w of mixed nuts); and (3) low-fat control diet. A 137-item FFQ was used to assess MedDiet adherence at baseline and after one year of intervention. Atherothrombosis biomarkers levels were quantified by enzyme-linked immunosorbent assay (ELISA).
Becerra-Tomás, Nerea et al., 2021 [44]	CSS	Spain	AMC	Elders	6475	A—55–75 years G—52.7% men BMI = 27–40 kg/m^2^	A 143-item FFQ was used to assess fruit and fruit juice consumption; a 17-item questionnaire was used to evaluate energy-reduced MedDiet adherence; sociodemographic and lifestyle variables were collected.
Jalilpiran, Yahya et al., 2020 [45]	CSS	Iran	HC	Elders	357	A— ≥ 60 years G—men	A 168-item semiquantitative FFQ was used to evaluate dietary intake. MedDiet and Dietary Approach to Stop Hypertension (DASH) dietary scores were calculated. Anthropometric measures, biochemical parameters, and overall characteristics were also collected.
Kanerva, Noora et al., 2014 [46]	Cohort study (CS)	Finland	AMC	Adults	6490	A—25–74 years G—53% women	Dietary intake was measured through a 130-item FFQ to calculate Baltic Sea Diet Score (BSDS). Anthropometric measures and leptin, adiponectin, tumor-necrosis factor alpha (TNF-α), interleukin-6 (IL-6), and high-sensitivity (hs)-CRP concentrations were assessed.
Khalatbari-Soltani, Saman et al., 2020 [47]	PCS	Switzerland	AMC	Adults	2288	A—55.8 ± 10.0 years G—65.4% women	Dietary intake was accessed by a 97-item FFQ to predict the MedDiet score; Fatty liver index (FLI) score was obtained through a logistic function, including BMI, WC, fasting triglycerides, and gamma-glutamyl transferase (GGT) levels, and Non-alcoholic fatty liver disease (NAFLD) liver fat score was calculated based on the presence of metabolic syndrome (MetS), T2D, fasting concentrations of insulin, Aspartate aminotransferase (AST), and the AST/ Alanine aminotransferase (ALT) ratio.
Kondo, Keiko et al., 2014 [48]	Prospective Non-randomized study (PNRS)	Japan	AMC	Adults	17	A—35–60 years G—82.4% men BMI ≥ 25 kg/m^2^	After 2–3 weeks, participants obtained a meal consisting of high fiber and low fat (30 kcal/kg of ideal BW), 3 x/day for 8 weeks, followed by a normal diet for 24 weeks. Insulin, glucose, glycated haemoglobin (HbA1c), lipids, hs-CRP, tissue plasminogen activator-1 (TPAI-1), fibrinogen, leptin, adiponectin, blood pressure (BP), BW, and WC were measured.
Martens, Remy J. H. et al., 2020 [49]	CSS	The Netherlands	AMC	Adults	2961	A—59.8 ± 8.2 years G—51% men	Sodium and potassium concentrations were obtained through urine samples, and high-sensitivity cardiac troponin T (hs-cTnT), high-sensitivity cardiac troponin I (hs-cTnI), and N-terminal pro-B-type natriuretic peptide (NT-proBNP) concentrations were measured in stored frozen serum samples.
Martínez-Lapiscina, Elena H. et al., 2014 [50]	RCT	Spain	HC	Elders	522	A—55–80 years (men) and 60–80 (women) D—T2D or CVD risk	Participants were allocated to one of these diets: two MedDiets (supplemented with either (1) extra-virgin olive oil or (2) nuts), or (3) a low-fat diet. After 6.5 years of intervention, they were assessed using the MMSE and the Clock Drawing Test (CDT). The CR1-rs3818361, CLU-rs11136000, PICALM-rs3851179, and Apolipoprotein E (ApoE) genes were genotyped in these participants.
Mofrad, Manije D. et al., 2019 [51]	CSS	Iran	AMC	Elders	362	A—60–80 years G—men	Diet was assessed using a 168-item FFQ. Elderly dietary index (EDI) adherence was calculated based on the modified MyPyramid for older adults. Anthropometric values, biochemical parameters, and BP were measured. The relationships between EDI tertiles and CVD risk factors were investigated using multivariate logistic regression.
Mujica-Parodi, Lilianne R. et al., 2020 [52]	CS	USA	AMC	Adults	42	A—18–88 years G—52.4% women BMI < 30 kg/m^2^	Metabolic neuroimaging datasets; Magnetic resonance imaging (MRI) acquisition and processing; spatial navigation and motor tasks; functional (fMRI) network analyses.
Neth, Bryan J. et al., 2020 [53]	COS	USA	AMC	Adults	20	A—50–80 years G—75% women D—MCI risk	Participants consumed either (1) modified Mediterranean-ketogenic diet (MMKD) or (2) American Heart Association Diet (AHAD) (control), for 6 w. Before diet randomization and after each diet, baseline cognitive status, lumbar puncture (LP), MRI, and metabolic profiles were executed.
Paoli, Antonio et al., 2011 [54]	PNRS	Italy	AMC	Adults	106	A—18–65 years G—82.1% women BMI ≥ 25 kg/m^2^	Participants received a Ketogenic Mediterranean with phytoextracts (KEMEPHY) for 6 w. Weight and TC, triglycerides, HDL-C, LDL-C, glucose, blood urea nitrogen (BUN), uricemia, VES, creatinine, ALT, AST, GGT levels were measured.
Bhanpuri, Nasir H. et al., 2018 [55]	PCS	USA	AMC	Adults	349	A—54 ± 8 years G—65.1 ± 3.2% women BMI = 25–30 kg/m^2^ D—T2D	Continuous care intervention (CCI): health coach and medical provider; Usual care (UC): independently recruited to path T2D progression; circulating biomarkers, BP, carotid intima media thickness (cIMT), multi-factorial risk scores and medication use were examined.
Schönknecht, Yannik B. et al., 2020 [56]	RCT	Germany	UH	Elders	60	A—60–80 years G—56.7% men BMI—27–34.9 kg/m^2^	Participants consumed three different isoenergetic meals: (1) Western diet-like high-fat (WDHF), (2) Western diet-like high-carbohydrate (WDHC), and (3) MedDiet. Blood samples were collected at fasting and between 1 and 5 h postprandially. Lipid and glucose metabolism parameters, inflammation, and oxidation levels, and antioxidant status were examined
Song, Xiaoling et al., 2016 [57]	RCT	USA	AMC	Adults	102	A—21–79 years G—51% men BMI—19.2–35.5 kg/m^2^	Participants were allocated three different diets for 6 w: (1) eucaloric moderate-fat diet, (2) eucaloric low-fat diet, and (3) low-fat diet with a 33% caloric deficit (“low-calorie low-fat diet). Plasma CRP, IL-6, leptin, total adiponectin, and soluble tumour necrosis factor receptors I & II (sTNFRI and -II) concentrations were assayed by ELISA.
Tiainen, A-MK. et al., 2012 [58]	CSS	Finland	UH	Adults	1942	A—57–70 years	LTL was measured by qPCR. A semiquantitative 12-item FFQ was used to evaluate the diet.
Uusitupa, M. et al., 2013 [59]	RCT	Denmark, Finland, Iceland and Sweden	AMC	Adults	166	A—30–65 years G—67% women BMI—27–38 kg/m^2^	Participants were randomized to two different diets for 18–24 w: (1) control diet or (2) healthy nordic diet. Biochemical and anthropometric measurements were collected.
Yousefi, Reyhaneh et al., 2020 [60]	RCT	Iran	AMC	Adults	40	A—20–50 years G—82.5% women BMI—25–40 kg/m^2^	Participants adhere to restricted-calorie diet (RCD) and received 300 mg/d of (1) grape seed extract (GSE) capsules or (2) placebo capsules for 12 weeks. Anthropometric and biochemical parameters dietary intake were evaluated.
Yubero-Serrano, Elena M. et al., 2012 [61]	RCT	Spain	UH	Elders	20	A— ≥ 65 years G—50% men BMI—20–40 kg/m^2^	Three different diets during the 4 weeks, each: (1) MedDiet, (2) Med + CoQ diet, and (3) SFA diet. p65, Inhibitor of nuclear factor kappa-B kinase subunit beta (IKK-β), Nuclear factor of kappa light polypeptide gene enhancer in B-cells inhibitor, alpha (IkB-α), Matrix metallopeptidase 9 (MMP-9), interleukin-1β (IL1-β), c-Jun N-terminal kinase-1 (JNK-1), x-box–binding protein-1 (sXBP-1), calreticulin (CRT), and glucose-regulated protein 78 kDa (BiP-Grp78) mRNAs levels were analyzed.
Boccardi, Virginia et al., 2013 [62]	CS	Italy	AMC	Elders	217	A— ≥ 65 years G—53% men BMI—25.86 ± 1.4 kg/m^2^	Association among TL, telomerase activity (TA), and MedDiet adherence was studied. Participants were divided according to MedDiet score (MDS) in low adherence (MDS < 3), medium adherence (MDS 4–5) and high adherence (MDS > 6). LTL was measured by qPCR and TA by a PCR-ELISA protocol.
Bonaccio, Marialaura et al., 2021 [63]	PCS	Italy	AMC	Adults	22,475	A ≥ 35 years G—53.4% women	A 188-item FFQ was used to assess food intake. The NOVA classification defined UPF, and those intakes were categorized as quartiles of the ratio (%) of UPF (g/d) to total food consumed (g/d).
Cassidy, Aedín et al., 2010 [64]	PCS	USA	UH	Adults	2284	A—30–55 years G—women	LTL was measured by qPCR. A questionnaire was used to examine anthropometric data, diet, and lifestyle.
Chou, Yi-Chun et al., 2019 [65]	PCS	Taiwan	UH	Elders	436	A— ≥ 65 years G—53% women BMI—23.8 ± 2.9 kg/m^2^	The modified Alternative Healthy Eating Index (mAHEI) was used to assess diet quality, which was calculated from a 44-item FFQ at baseline, and vegetable variety was derived from the diet diversity score (DDS). Montreal Cognitive Assessment—Taiwanese version (MoCA-T) (global cognition) and Wechsler Memory Scale-Third edition (WMS-III) (domain cognition) were used to assess global and domain-specific cognition (logical memory and attention domains).
Crous-Bou, Marta et al., 2014 [66]	CS	USA	AMC	Adults	4676	A—42–70 years G—women	The relationship between relative TL in peripheral blood leukocytes measured by qPCR and the alternate MDS calculated from self-reported dietary data.
do Rosario, Vinicius A. et al., 2020 [67]	RCT	Australia	AMC	Adults	16	A ≥ 55 years G—81.3 women BMI ≥ 25 kg/m^2^	High fat high energy (HFHE) meal along with 250 mL of: (1) anthocyanins-rich Queen Garnet plum juice (intervention) or (2) apricot juice (control). Blood samples and BP measures were collected at baseline, 2 h, and 4 h following the meal. Vascular and microvascular function were evaluated at baseline and 2 h after the meal.

**Table 2:** Summary of the included studies’ results of outcomes related to molecular output and related biomarkers.

Authors (Year)	Main Results	Biomarkers and Outcomes
Alonso-Pedrero, Lucia at al., 2020 [32]	Higher consumption of UPF (*>*3 servings/d) presented higher risk of having shorter telomeres in an elderly Spanish population.	Participants with >3 servings/day of UPF consumption: higher short telomere risk (*p* = 0.032), higher family history of CVD (*p* = 0.045), and diabetes and dyslipidemia prevalence (*p* = 0.014); higher consumption of fats, SFAs, sodium, sugar-sweetened beverages (SSBs), fast food, and processed meat (*p* < 0.001), PUFAs (*p* = 0.011), dietary cholesterol (*p* = 0.008); less adherence to the MedDiet (*p* < 0.001).
Baba, Yoshitake et al., 2020 [33]	Intake of 336.4 mg of Green Tea Catechins (GTC) promoted working memory in adults.	GTC: significantly lower commission errors on the CPT (*p* = 0.004), after a single dose; significantly lower correct response time on the 4-part CPT (FPCPT) (*p* = 0.012).
Fernández-Real, José Manuel et al., 2012 [34]	Consumption of MedDiet + VOO for 2 years increased (serum osteocalcin) and (P1NP), indicating bone-protective effects.	MedDiet + VOO: TOC concentrations increased (*p* = 0.007), P1NP levels increased (*p* < 0.01); consumption of olives: positively associated with both baseline total osteocalcin (*p* = 0.02) and 2 year (osteocalcin) (*p* = 0.04).
Fortin, A. et al., 2018 [35]	MedDiet and low-fat diet in patients with T1D and MetS could help with weight loss, with no significant changes in anthropometric and metabolic parameters between regimens.	BMI, WC, weight, and triglycerides: decreased overtime with both diets (*p* < 0.05).
Fretts, Amanda M. et al., 2016 [36]	Processed meat, but not unprocessed red meat consumption, was linked to a shorter LTL.	Processed meat: inverse correlation with LTL (*p* = 0.009), after adjustment for potential mediators, including SBP, LDL-C, fibrinogen, and BMI.
García-Calzón, Sonia at al., 2015 [37]	Diet, through proinflammatory or anti-inflammatory pathways, could be a fundamental predictor of telomere length.	DII score ^3^: inverse significant association with TL (*p* = 0.001)
González-Guardia, Lorena et al., 2015 [38]	MedDiet + CoQ promotes urine metabolites excretion, reducing oxidative stress. Metabolites excreted after SFA diet are linked to increased oxidative stress.	MedDiet + CoQ: higher hippurate urine levels and reduced phenylacetylglycine levels (*p* < 0.05); inversely related to Nrf2 and thioredoxin (Trx) (*p* = 0.004), superoxide dismutase 1 (SOD-1) (*p* = 0.03) and gp91phox subunit of nicotinamide adenine dinucleotide phosphate (NADPH) oxidase gene expression (*p* = 0.039); SFA diet: phenylacetylglycine excretion was negatively related to CoQ (*p* = 0.039) and positively correlated with isoprostane urinary levels (*p* = 0.013).
Gu, Yian et al., 2015 [39]	Among whites, greater adherence to a MedDiet was significantly connected with longer LTL. In addition, eating a diet rich in vegetables and poor in meat, dairy, or cereal might contribute to longer LTL.	MedDiet adherence: higher LTL in whites (*p*-trend = 0.02); consumption of vegetables and cereals: increased LTL (*p* = 0.002 and *p* = 0.003); consumption of reduced dairy and meat intake: increased LTL (*p* = 0.05 and *p* = 0.004).
Guallar-Castillón, Pilar et al., 2012 [40]	SEAD may prevent myocardial infarction by lowering inflammation markers and reducing triglycerides, insulin, insulin resistance, and SBP.	Higher SEAD adherence: lower plasma CRP, insulin, homeostasis model assessment-insulin resistance (HOMA-IR), urine albumin, and SBP (*p*-trend < 0.001), triglycerides (*p*-trend = 0.012), urine albumin/creatinine ratio (*p*-trend < 0.034).
Gutierrez-Mariscal, Francisco M. et al., 2012 [41]	MedDiet protects DNA from oxidative damage and CoQ supplementation enhances this protection, lowering p53 activation. On the other hand, SFA diet potentiate oxidative stress and p53 stabilization.	Med + CoQ diet: increase of fasting plasma (CoQ) and postprandial (2 h) plasma [CoQ] (*p* < 0.001 and *p* = 0.018); decrease of plasma (8-OHdG) (*p* < 0.0001) and after postprandial period (*p* = 0.026), of p53 postprandial levels (*p* < 0.05), of nuclear *p*-p53 (Ser20) postprandial levels (*p* = 0.0013), of NM-p53 postprandial (*p* < 0.05) and of CM-p53 postprandial levels (*p* = 0.046); MedDiet: decrease of CM-p53 postprandial levels (*p* = 0.043) and increase of *mdm2* mRNA levels (*p* < 0.05); SFA diet: higher fasting plasma concentrations of TC (*p* < 0.001), LDL-C (*p* = 0.013), ApoB (*p* = 0.017), Apolipoprotein A1 (ApoA1) (*p* = 0.002) and p53 mRNA levels (*p* = 0.047);
Gutierrez-Mariscal, Francisco M. et al., 2014 [42]	In comparison to the harmful activity of an SFA diet, which initiates the p53-dependent DNA repair mechanism, the MedDiet diet and MedDiet + CoQ10 have beneficial effects on DNA damage.	Med + CoQ: lower mRNA *Gadd45a*, mRNA *Gadd45b,* mRNA *Ogg1*, nuclear APE-1/Ref-1 protein level, mRNA *DNA polβ*, and mRNA *XPC* (*p* = 0.044, *p* = 0.027, *p* = 0.048, *p* = 0.038, *p* = 0.041 and *p* = 0.019, respectively).
Hernáez, Álvaro et al., 2020 [43]	The MedDiet improved atherothrombosis biomarkers (HDL, fibrinogen, and Non-esterified fatty acids (NEFA) levels) in high cardiovascular risk individuals.	Adherence to MedDiet: increased activity of platelet activating factor acetylhydrolase (PAF-AH) in HDLs (adjusted difference: +7.5% (0.17; 14.8) and HDL-bound 𝛼1-antitrypsin levels (adjusted difference: −6.1% [−11.8; −0.29]; reduced fibrinogen (adjusted difference: −9.5% (−18.3; −0.60) and NEFA concentrations (adjusted difference: −16.7% (−31.7; −1.74)).
Becerra-Tomás, Nerea et al., 2021 [44]	In older adults with MetS, increased total fruit consumption is linked with lower WC, plasma glucose and LDL-C levels, as well as higher SBP and DBP. Total and natural fruit juice consumption was associated with reduced WC and glucose levels.	Higher total fruit consumption: significantly reduction in WC and glucose (*p* = 0.01) and LDL-C (*p* < 0.01); significantly increase DBP (*p* < 0.01); higher total fruit juice consumption: significantly reduces WC and glucose (*p* < 0.01); higher consumption of orange fruits (increase in SBP and DBP (*p* < 0.01)); green fruits (decrease in glucose (*p* = 0.01) and increase in HDL-C (*p* = 0.01)); red/purple fruits (decrease in glucose (*p* = 0.01)); white fruits (decrease in BMI and WC (*p* < 0.01)).
Jalilpiran, Yahya et al., 2020 [45]	Inverse correlation between the DASH and MedDiet patterns and several cardiovascular risk factors.	Greater adherence to MedDiet: lower WC, triacylglycerol, hs-CRP, fibrinogen, and higher HDL-C (*p* < 0.05); lower DBP (*p* = 0.01) and fibrinogen levels (*p* < 0.001); Greater adherence to DASH: lower fibrinogen (*p* < 0.05); reduced risk of high DBP (*p* < 0.001), insulin levels (*p* = 0.001), hs-CRP (*p* = 0.009), and fibrinogen (*p* < 0.001).
Kanerva, Noora et al., 2014 [46]	Lower hs-CRP levels are due to the Baltic Sea diet.	BSDS: inverse association with hs-CRP (*p* < 0.01), contributed mainly by high intake of Nordic fruits and cereals, low intake of red and processed meat, and moderate intake of alcohol (*p* < 0.05).
Khalatbari-Soltani, Saman et al., 2020 [47]	Adherence to the MedDiet decreased hepatic steatosis risk based on the FLI, in addition to the existing evidence of reducing CVD risk. When different parameters for determining the NAFLD score were used, no connection was found.	Adherence to MedDiet: lower risk of hepatic steatosis based on FLI (*p*-trend < 0.006), after adjustment for BMI (*p*-trend = 0.031) and after adjustment of BMI and WC (*p*-trend = 0.034).
Kondo, Keiko et al., 2014 [48]	Treatment with a high-fiber, low-fat diet for 8 weeks effectively improved periodontal disease markers and metabolic profiles, at least in part, by mechanisms effects other than caloric restriction.	High-fiber, low-fat diet: significantly reduced probe depth (PD), Clinical attachment loss (CAL), bleeding on probing (BOP), and Gingival crevicular fluid (GCF) (*p* < 0.005), and showed improvement of BW, HbA1c (*p* < 0.0001), and hs-CRP (*p* = 0.038).
Martens, Remy J. H. et al., 2020 [49]	24 h urinary sodium excretion (UNaE) was not connected with the examined cardiac biomarkers; lower 24 h urinary potassium excretion (UKE) was nonlinearly linked with higher hs-cTnT and NT-proBNP.	Diets rich in Potassium: lower hs-cTnT (*p* = 0.023) and NT-proBNP (*p* = 0.005).
Martínez-Lapiscina, Elena H. et al., 2014 [50]	The preventive impact of MedDiet may be larger for patients with a favorable genetic profile because it regulates the effect of genetic risk factors on cognition.	MedDiet: beneficial effect in *CLU* gene *rs11136000* variant carrying the T minor allele in MMSE test (*p* < 0.001) and CDT score (*p* = 0.001), in *CR1* gene *rs3818361* variant without the A minor risk allele in MMSE test (*p* = 0.001) and CDT score (*p* = 0.006); in *PICALM rs3851179* polymorphism with at least one T minor allele in CDT score (*p* = 0.005); and in non-APOE4 carriers in MMSE test (*p* = 0.007) and CDT (*p* < 0.001)
Mofrad, Manije D. et al., 2019 [51]	Higher EDI was associated with lower risk of being overweight or obese, as well as having LDL-C levels. However, in elderly men, there was no significant association between EDI and other CVD risk factors.	Highest tertile of EDI: higher consumption of fruits, vegetables, fish, olive oil, bread, cereal, and dairy products (*p* < 0.05); EDI: associated with higher intakes of carbohydrates, SFA, PUFAs, monounsaturated fatty acids (MUFAs), cholesterol, folate vitamin B1, vitamin B6, vitamin A, vitamin C, potassium, and magnesium (*p* < 0.05); Higher EDI: lower weight, BMI, WC, serum insulin, HOMA-IR, fibrinogen, ALT, AST, and DBP (*p* < 0.05); higher fasting blood sugar (FBS), HDL-C, TC levels, and quantitative insulin sensitivity check index (QUICKI) (*p* < 0.05).
Mujica-Parodi, Lilianne R. et al., 2020 [52]	Destabilization of brain networks may be an early sign of hypometabolism, which is linked to dementia. Dietary interventions that result in ketone utilization increase available energy and, as a result, may have the potential to protect the aging brain.	KD: decreased destabilization of brain network (DBN) (*p* < 0.001); higher amplitude for low-frequency fluctuations (ALFF) (*p* < 0.001); cognitive acuity: declined with age (*p* < 0.001); network switching: inverse association with ALFF (*p* < 0.001).
Neth, Bryan J. et al., 2020 [53]	MMKD may help prevent cognitive decline in adults at risk of Alzheimer’s disease (AD) risk, by improving cerebral spinal fluid (CSF) AD biomarker profile, peripheral lipid and glucose metabolism, cerebral perfusion and cerebral ketone body uptake.	MMKD: higher fasting ketone body levels (*p* = 0.008), mainly in subjective memory complaints (SMC) group (*p* = 0.015); reduced very low-density lipoprotein cholesterol (VLDL-C) levels and triglycerides (*p* = 0.02); increased CSF Aβ42 (*p* = 0.04) and decreased tau levels in mild cognitive impairment (MCI) group (*p* = 0.007); increased cerebral perfusion, mainly in MCI group (*p* < 0.05) and cerebral ketone body uptake (11C-acetoacetate (*p* = 0.02); AHAD: decreased tau levels in MCI group (*p* = 0.02).
Paoli, Antonio et al., 2011 [54]	The KEMEPHY diet resulted in weight and WC loss, as well as improvements in cardiovascular risk markers.	KEMEPHY diet: reduction in BMI, BW, % fat mass, WC, TC, LDL-C, triglycerides, and blood glucose (*p* < 0.0001); increase in HDL-C (*p* < 0.0001).
Bhanpuri, Nasir H. et al., 2018 [55]	After a year, CCI improved the majority of biomarkers of CVD risk in T2D patients. The increase in LDL-C seems to be restricted to the large LDL subfraction. LDL particle size increased, while total LDL-P and ApoB remain unchanged, and inflammation and BP decreased.	Decrease in weight, ApoB/ApoA1 ratio, triglycerides, triglycerides/HDL-C ratio, large very low-density lipoprotein particle (VLDL-P), small LDL-P, BP, hs-CRP, white blood count (WBC), 10-year Atherosclerotic cardiovascular disease (ASCVD) risk score, antihypertensive medication (AHM) use (*p* < 0.001); increase in ApoA1, LDL-C, HDL-C, LDL-P size, and large HDL-P (*p* < 0.001).
Schönknecht, Yannik B. et al., 2020 [56]	A high-energy meal caused hyperglycemia, hyperlipemia, and a decrease in antioxidant markers, whereas the MedDiet had a positive effect on glycemic, insulinemic, and lipemic responses.	WDHC: increased glucose (*p* = 0.002) and insulin levels (*p* < 0.001), compared with other meals WDHF: increased triglycerides levels and higher NEFA (*p* < 0.001), compared with other meals MedDiet: higher vitamin C levels (*p* < 0.001), compared with other meals.
Song, Xiaoling et al., 2016 [57]	Moderate weight loss had little effect on systemic inflammation in relatively healthy adults. A lower dietary fat and higher carbohydrate content had little impact on systemic inflammation measures but significantly reduced adiponectin concentrations when compared to a moderate-fat diet.	Low-calorie, low-fat,(LCLF) diet: greater reductions in weight, fat mass and fasting leptin levels (*p* < 0.001), compared to other diets; reduced adiponectin (*p* = 0.008), compared to low-fat diets; adiponectin: tend to increase with weight loss (*p* = 0.051).
Tiainen, A-MK. et al., 2012 [58]	Fat intake is inversely associated with LTL whereas vegetable intakes were positively associated with LTL.	Vegetable intake: positive association with LTL (*p* = 0.05) in women, after adjustments. Total fat, SFAs, and butter intake: inverse correlation with LTL (*p* = 0.04, *p* = 0.01 and *p* = 0.04).
Uusitupa, M. et al., 2013 [59]	Healthy Nordic diet improved lipid profile and reduced low-grade inflammation.	Healthy Nordic diet: lower non-HDL-C (*p* = 0.04), LDL-C/HDL-C ratio (*p* = 0.046), ApoB/ApoA1 ratio (*p* = 0.025); control diet: increased interleukin-1 receptor antagonist (IL-1 Ra) (*p* = 0.00053), related with saturated fats and magnesium^+^ intake (*p* = 0.049 and *p* = 0.012).
Yousefi, Reyhaneh et al., 2020 [60]	When combined with a calorie-restricted diet, daily consumption of 300 mg GSE improved LDL-C, HDL-C, visceral adiposity index (VAI), and atherogenic index of plasma (AIP) and helps to ameliorate some CVD risk factors in obese or overweight individuals.	GSE: significantly increase in HDL-C and HDL-C/LDL-C at w 12 (*p* = 0.01 and 0.003, respectively) and significantly decrease in LDL-C (*p* = 0.04), compared to placebo; significantly decreased VAI, AIP, TC and triglycerides compared to baseline (*p* =0.04, *p* = 0.02, *p* =0.01 and *p* = 0.02, respectively).
Yubero-Serrano, Elena M. et al., 2012 [61]	The anti-inflammatory effect of a MedDiet rich in olive oil and exogenous CoQ supplementation has an additive effect in aged men and women, regulating the inflammatory response and ER stress, indicating that a MedDiet + CoQ is helpful for healthy aging.	Med + CoQ: higher fasting plasma (CoQ) (*p* < 0.001) and plasma CoQ levels compared with the Med and SFA diets (*p* = 0.018 and *p* = 0.032), increase in IkB-α mRNA levels compared with the SFA diet (*p* = 0.028), decrease in IKK-β, p65 and IL-1β mRNA levels compared with the other diets (*p* = 0.010; *p* = 0.008 and *p* = 0.012; *p* = 0.011). MedDiet: lower p65, IKK-β, MMP-9 and IL-1β mRNA levels compared with the SFA diet (*p* = 0.033, *p* = 0.034; *p* = 0.034; *p* = 0.029), higher levels of IkB-α mRNA (*p* = 0.018). SFA diet: higher MMP-9 (*p* = 0.008 and *p* = 0.032), IL-1b (*p* = 0.017), JNK-1 (*p* = 0.037), sXBP-1 (*p* = 0.033 and *p* = 0.008), CRT (*p* = 0.031) and BiP/Grp78 (*p* = 0.021) mRNA levels compared with Med and Med + CoQ diets.
Boccardi, Virginia et al., 2013 [62]	Lower telomere shortening and higher Peripheral blood mononuclear cells (PBMCs) TA may play a role in lifespan and, more importantly, health span in populations consuming traditional MedDiet.	LTL: shorter with age (*p* < 0.001) and positive correlation with TA (*p* = 0.028), higher in women (*p* < 0.001) and differ according to smoking status (*p* < 0.001); negatively correlated with IS (*p* < 0.001) and nitrotyrosine (*p* = 0.011). PBMC TA: negatively correlated with both inflammation score (IS) (*p* = 0.048) and nitrotyrosine levels (*p* = 0.022); MDS ≥ 6: longer TL (*p* = 0.003) and higher TA (*p* = 0.013); lower plasmatic levels of CRP (*p* = 0.018), IL-6 (*p* = 0.010), TNF-α (*p* = 0.021) and nitrotyrosine (*p* = 0.009); IS: positively correlated with nitrotyrosine levels (*p* < 0.001).
Bonaccio, Marialaura et al., 2021 [63]	Higher levels of UPF were linked to increased risk of CVD and all-cause mortality, partly due to its high dietary content of sugar.	Intake of UPF: lower adherence to the MedDiet and intake of fiber (*p* < 0.001); higher energy intake, fat, sugar, dietary cholesterol, and Na^+^ (*p* < 0.001); increased risks of CVD mortality (HR: 1.58; 95% CI: 1.23, 2.03), death from ischemic heart disease (IHD)/cerebrovascular disease (HR: 1.52; 95% CI: 1.10, 2.09), and all-cause mortality (HR: 1.26; 95% CI: 1.09, 1.46).
Cassidy, Aedín et al., 2010 [64]	LTL, which is a putative biomarker of chronic disease risk, is associated with body composition and dietary factors.	Fiber intake (cereal fiber and whole grains) and vitamin D: higher LTL (*p* = 0.006, *p* = 0.01 and *p* = 0.01); LTL: inversely correlated with age (*p* < 0.0001), BMI (*p* = 0.005), WC (*p* = 0.009), weight (*p* = 0.004) and linoleic acid (*p* = 0.0009) and total fat intake (*p* = 0.003), including MUFAs and PUFAs (*p* = 0.006 and *p* = 0.0008).
Chou, Yi-Chun et al., 2019 [65]	In older adults, a high-quality diet containing a variety of vegetables was linked to a lower incidence of cognitive decline.	High diet quality with high vegetable diversity: lower risk of global cognitive decline (*p*-trend = 0.03) and of decline of attention domain (*p*-trend = 0.049); lower risk of global cognitive decline (*p*-trend = 0.03) in elders.
Crous-Bou, Marta et al., 2014 [66]	Longer telomeres were associated with greater adherence to the MedDiet. These results further support the benefits of adhering to this diet in terms of promoting health and longevity.	MedDiet score: proportional with TL (*p* = 0.016); higher in women with lower BMI (*p* = 0.01), who smoked less, had higher intake of total energy, were more physically active; higher with vegetables, fruits, grains, fish, nuts, and total fat intake, as well as lower meat intake (*p* < 0.001); TL: longer in younger women (*p* < 0.001); shorter in women who smoked more (*p* = 0.02); LTL: longer with AHEI (*p* = 0.02).
do Rosario, Vinicius A. et al., 2020 [67]	In overweight older individuals, fruit-based anthocyanins attenuated the potential negative postprandial effects of a HFHE challenge on vascular and microvascular function, as well as inflammation biomarkers.	Anthocyanin: higher postprandial flow mediated dilation (FMD) and post-occlusive reactive hyperaemia maximum perfusion (PORHmax) (*p* < 0.05), after 2 h; lower CRP (*p* < 0.05) and trend to lower IL-6 (*p* = 0.075), after 4 h.

## Data Availability

Not applicable.

## Supplementary Materials

The following are available online at https://www.mdpi.com/article/10.3390/nu14030554/s1, Table S1: Quality assessment of the selected articles.
