# Supplementary material for: The Effect of Nutrition on Aging—A Systematic Review Focusing on Aging-Related Biomarkers
Source: Nutrients. 2022 Jan 27;14(3):554. doi: 10.3390/nu14030554 (PMC8838212; doi:10.3390/nu14030554)
Supplement: Supplementary file 1 [file nutrients-14-00554-s001.zip › nutrients-1547299-supplementary.pdf]

Table S1 – Quality assessment of the included studies.

Author (year)	Allocation of study groups	Unit of allocation	Baseline differences	Objectivity of the outcome	Completeness of follow-up	Total score
Alonso-Pedrero, Lucia at al., 2020 [32]	1	0	2	2	1	**6**
Baba, Yoshitake et al., 2020 [33]	2	2	1	0	2	**7**
Fernández-Real, José Manuel et al., 2012 [34]	2	1	2	1	2	**8**
Fortin, A. et al., 2018 [35]	2	1	1	1	2	**7**
Fretts, Amanda M. et al., 2016 [36]	0	1	2	2	0	**5**
García-Calzón, Sonia et al., 2015 [37]	2	1	2	1	2	**8**
González-Guardia, Lorena et al., 2015 [38]	2	0	1	0	0	**3**
Gu, Yian et al., 2015 [39]	1	1	2	2	1	**7**
Guallar-Castillón, Pilar et al., 2012 [40]	1	1	2	1	2	**7**
Gutierrez-Mariscal, Francisco M. et al., 2012 [41]	2	1	1	0	2	**6**
Gutierrez-Mariscal, Francisco M. et al., 2014 [42]	2	1	1	0	2	**6**
Hernáez, Álvaro et al., 2020 [43]	2	2	2	0	2	**8**
Becerra-Tomás, Nerea et al., 2021 [44]	1	1	2	1	2	**7**
Jalilpiran, Yahya et al., 2020 [45]	1	2	2	1	2	**8**
Kanerva, Noora et al., 2014 [46]	2	1	2	1	0	**6**
Khalatbari-Soltani, Saman et al., 2020 [47]	1	1	2	2	0	**6**
Kondo, Keiko et al., 2014 [48]	0	2	0	1	0	**3**
Martens, Remy J. H. et al., 2020 [49]	1	2	2	1	1	**7**
Martínez-Lapiscina, Elena H. et al., 2014 [50]	2	2	2	2	0	**8**
Mofrad, Manije D. et al., 2019 [51]	1	1	2	1	0	**5**
Mujica-Parodi, Lilianne R. et al., 2020 [52]	1	0	1	0	0	**2**
Neth, Bryan J. et al., 2020 [53]	2	1	1	1	1	**6**
Paoli, Antonio et al., 2011 [54]	0	1	1	2	1	**5**
Bhanpuri, Nasir H. et al., 2018 [55]	0	1	2	0	1	**4**
Schönknecht, Yannik B. et al., 2020 [56]	2	1	1	0	2	**6**
Song, Xiaoling et al., 2016 [57]	2	1	2	0	2	**7**

Author (year)	Allocation of study groups	Unit of allocation	Baseline differences	Objectivity of the outcome	Completeness of follow-up	Total score
Tiainen, A-MK. et al., 2012 [58]	1	0	1	0	2	**4**
Uusitupa, M. et al., 2013 [59]	2	2	2	2	1	**9**
Yousefi, Reyhaneh et al., 2020 [60]	2	1	2	2	1	**8**
Yubero-Serrano, Elena M. et al., 2012 [61]	2	1	1	0	2	**6**
Boccardi, Virginia et al., 2013 [62]	1	1	1	0	2	**5**
Bonaccio, Marialaura et al., 2021 [63]	0	0	2	1	2	**5**
Cassidy, Aedín et al., 2010 [64]	0	0	2	0	0	**2**
Chou, Yi-Chun et al., 2019 [65]	1	2	2	0	1	**6**
Crous-Bou, Marta et al., 2014 [66]	0	0	2	2	0	**4**
do Rosario, Vinicius A. et al., 2020 [67]	2	1	1	2	2	**8**
						**Median**
						**6**
